# Hyperbaric oxygen-induced acute lung injury: A mouse model study on pathogenic characteristics and recovery dynamics

**DOI:** 10.3389/fphys.2024.1474933

**Published:** 2024-10-18

**Authors:** Shu Wang, Hong Chen, Zhi Li, Guangxu Xu, Xiaochen Bao

**Affiliations:** ^1^ Department of Rehabilitation Medicine, The First Affiliated Hospital of Nanjing Medical University, Nanjing, China; ^2^ Department of Diving and Hyperbaric Medicine, Naval Medical Center, Shanghai, China; ^3^ Cadre Diagnosis and Treatment Department, The General Hospital of the People’s Liberation Army, Beijing, China; ^4^ The First Affiliated Hospital of Nanjing Medical University, Nanjing, China

**Keywords:** hyperbaric oxygen (HBO), acute lung injury (ALI), hyperoxic acute lung injury (HALI), reactive oxygen species (ROS), pulmonary oxygen toxicity (POT)

## Abstract

Oxygen is an essential substance for the maintenance of human life. It is also widely used in clinical and diving medicine. Although oxygen is crucial for survival, too much oxygen can be harmful. Excessive oxygen inhalation in a short period of time can lead to injury, and the lung is one of the main target organs. Acute lung injury (ALI) induced by hyperbaric oxygen (HBO) is notably more severe than that caused by normobaric oxygen, yet systematic research on such injury and its regression is scarce. In this study, two independent experiments were designed. In the first experiment, mice were exposed to 2 atmospheres absolute (ATA), ≥95% oxygen for 2, 4, 6, and 8 h. Changes in lung histopathology, inflammation and expression of chemokines, alveolar-capillary barrier, and 8-OHdG were detected before and after the exposure. In the second experiment, these parameters were measured at 0 h, 12 h, and 24 h following 6 h of exposure to 2 ATA of ≥95% oxygen. Research indicates that ALI induced by HBO is characterized histologically by alveolar expansion, atelectasis, inflammatory cell infiltration, and hemorrhage. At 2 ATA, significant changes in the alveolar-capillary barrier were observed after more than 95% oxygen exposure for 4 h, as evidenced by increased Evans blue (EB) extravasation (*p* = 0.0200). After 6 h of HBO exposure, lung tissue pathology scores, 8-OHdG levels, and inflammatory and chemotactic factors (such as Il6, CCL2, CCL3, CXCL5, and CXCL10), intercellular adhesion molecule 1 (ICAM1), and vascular cell adhesion molecule 1 (VCAM1) were significantly elevated. Compared to lung injury caused by normobaric oxygen, the onset time of injury was significantly shortened. Additionally, it was observed that these markers continued to increase after leaving the HBO environment, peaking at 12 h and starting to recover at 24 h, indicating that the peak of inflammatory lung injury occurs within 12 h post-exposure, with recovery beginning at 24 h. This contradicts the common belief that lung injury is alleviated upon removal from a high-oxygen environment. However, EB levels, which reflect damage to the alveolar-capillary barrier, and VE-Cadherin (VE-Cad), tight junction protein 1 (ZO-1), ICAM1, and VCAM1 remained significantly altered 24 h after leaving the HBO environment. This suggests that the alveolar-capillary barrier is the most sensitive and slowest recovering part of the lung injury induced by HBO. These findings can provide insights into the pathogenesis and progression of lung injury caused by HBO and offer references for identifying corresponding intervention targets.

## 1 Introduction

Oxygen is an essential substance for the maintenance of human life. It is also widely used in clinical and diving medicine. Critically ill patients need to continuously inhale high concentrations of oxygen to improve hypoxic conditions (Alva et al., 2023). Divers inhale high partial pressures of oxygen underwater, and high-pressure oxygen is also used in the treatment of decompression sickness (Bennett et al., 2010). The body’s inhalation of an excessive amount of oxygen can disrupt the balance of the oxidative-antioxidative system, causing damage to the organism (Taabazuing et al., 2014). Oxygen metabolizes in the body to produce oxygen free radicals, which are normally metabolized by the body’s antioxidative system. However, inhaling too much high concentration/high partial pressure oxygen can lead to an excessive accumulation of oxygen free radicals in the body, damaging DNA, proteins, and lipids (Alva et al., 2023).

The lung is exposed to higher oxygen tensions than any other organ and it is also the first organ to respond adversely to the toxic effects of oxygen. Therefore, the lung is one of the main target organs for oxygen toxicity. Prolonged exposure to normobaric oxygen results in widespread pulmonary damage with inflammation, accumulation of pleural fluid, respiratory failure, and death (Capellier et al., 1999). When oxygen pressures exceed 1 ATA, the development of lung injury is accelerated and worsened. Studies have shown that exposure to higher pressures of oxygen without seizures leads to a different pattern of lung damage compared to lower pressures (Demchenko et al., 2007). Our previous studies have demonstrated that the expression of cytokines, cellular apoptosis, and endothelial dysfunction were significantly increased in lung injury induced by hyperbaric oxygen (HBO) exposure compared with room air controls in rats (Bao et al., 2014) and mice (Bao et al., 2018). Excessive ROS leads to endothelial damage and inflammatory activation, resulting in increased expression of inflammatory cytokines and transcription factors in lung tissue (Dias-Freitas et al., 2016), such as tumor necrosis factor (Tnf), interleukin 1 beta (Il1b), interleukin 6 (Il6), and interleukin 10 (Il10) (Li et al., 2011; Entezari et al., 2014; Ning et al., 2020). Additionally, acute lung injury (ALI) continues to progress even 24 h after HBO exposure, and apoptosis may be involved in the pathology of ALI induced by hyperoxia (Han et al., 2018). Currently, there is limited research on the duration of HBO exposure that leads to ALI and the recovery progress of ALI after HBO exposure. It is important to determine both the occurrence and the recovery process of ALI induced by HBO, as this will provide a foundation for preventing ALI caused by HBO.

In this study, two independent experiments were designed. The first experiment is designed to understand ALI caused by different durations of HBO exposure. Mice were exposed to a hyperbaric environment with over 95% oxygen at 2 ATA for durations of 2, 4, 6, and 8 h. Changes in lung damage, alveolar-capillary barrier, inflammatory responses, and oxidative stress were detected after varying exposure times. The second experiment is designed to understand the progression and recovery of ALI after the termination of HBO exposure. The changes in the corresponding indicators in mouse lung tissues at 0, 12, and 24 h after removal from the HBO environment were examined to clarify the recovery process of ALI caused by HBO.

## 2 Materials and methods

### 2.1 Animals and ethical statement

7-8-week-old, male C57BL/6J mice were used in all experiments. Mice were housed in isolated cages and acclimatized to our housing facility for 1 week before experiments. All studies with mice were approved by the Naval Medical Center Animal Care and Use Committee (ethics number: NMC-202009).

### 2.2 HBO treatment procedure

**FIGURE 1:**
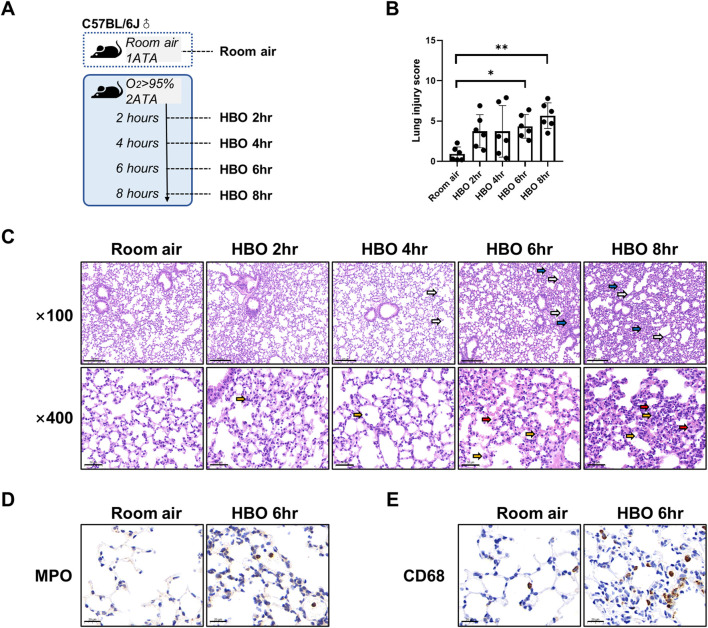
Lung histopathology in response to different durations of HBO exposure. **(A)** Experimental scheme. In the control group (Room air), mice were placed in the hyperbaric chamber at atmospheric pressure with air, while the experimental groups were exposed to 2ATA and ≥95% HBO for 2 h (HBO 2 h), 4 h (HBO 4 h), 6 h (HBO 6 h), and 8 h (HBO 8 h), respectively. **(B)** Assessment of lung injury scores for H&E-stained images, n = 6, ∗*p* ≤ 0.05, ∗∗*p* ≤ 0.01. **(C)** Representative H&E-stained images of mouse lung sections. Yellow arrows: infiltration of alveolar and interstitial inflammatory cells; red arrows: alveolar and interstitial hemorrhage; blue arrows: atelectasis; white arrows: alveolar dilation. Scale bars: 200 µm (100×) and 50 µm (400×). **(D–E)** Immunohistochemistry staining of lung tissues, MPO **(D)** and CD68 **(E)** was used to label neutrophils and macrophages, Scale bars: 20 µm.

Animal experiments were divided into two parts:

Part 1: 30 mice were randomly divided into 5 groups. The HBO groups were placed in a cylindrical HBO chamber with pre-placed sodalime at the bottom (Yantai Hongyuan Oxygen Industry Co., Ltd., Shandong, China). The lime at the bottom was placed to minimize water vapor and CO_2_ accumulation. Before pressurization, 100% medical oxygen was flushed through the chamber for 10 min to replace the air. Then, slow pressurization was applied to reach 0.2 megapascals for 10 min. Oxygen concentration was continuously monitored and maintained at ≥ 95%. The CO_2_ concentration was kept below 300 ppm. The condition of the mice was monitored by a camera located in the chamber and was recorded by a computer outside the chamber. After exposure to oxygen for 2, 4, 6, and 8 h at 0.2 MPa, mice were slowly decompressed to normal atmospheric pressure within 10 min. The control group (Room air group) was placed in the hyperbaric chamber at atmospheric pressure with air. After exposure, mice were immediately anesthetized by intraperitoneal injection of 1% pentobarbital sodium (0.1 g/kg body weight), the chest was exposed, and the lungs were collected after clearing the blood by perfusion with cold PBS via the right ventricle (Figure 1A).

Part 2: 24 mice were randomly divided into 4 groups. The control group (Room air group) was placed in the hyperbaric chamber under atmospheric pressure and exposed to ambient air. In HBO groups, mice were exposed to HBO for 6 h and then depressurized to normal pressure. Lung tissue was collected at 0 h (0 h), 12 h (12 h), and 24 h (24 h) after exposure (Figure 5A).

### 2.3 Vascular permeability assay

Vascular permeability in the lungs was assessed using the Evans blue (EB) assay (Wang et al., 2024). Mice were injected with 1% w/v EB solution at a dose of 40 mg/kg body weight intravenously, 45 min before sacrifice. To remove the intravascular dye, the mice were perfused with PBS through the right ventricle. The lungs were then flushed and homogenized using PBS (1 mL PBS/100 mg lungs). The EB was extracted from the homogenates by incubating and shaking overnight at 60°C in formamide (twice the volume of the homogenates). After centrifugation at 5000r for 30 min, the concentration of EB in the lung supernatants was quantified using wavelength spectrophotometric analysis at 620 nm. A standard curve was generated by diluting the EB serially. The concentration of extravasated EB (µg of EB per g lung) in the lung was calculated based on the standard curve.

### 2.4 Hematoxylin and eosin (H&E) staining and histological assessment of lung injury

For histological analysis, the left lungs of mice were fixed in 4% paraformaldehyde in PBS for 2 days, then paraffin-embedded and cut into 3 µm thick sections for H&E staining. Post H&E staining, panoramic images of the lung tissue were scanned, and 10 fields of ×400 view were randomly selected for lung injury scoring (Wang et al., 2020), with the average score taken. Within each field, lung injury was scored based on edema, inflammatory cell infiltration, hemorrhage, and atelectasis. Each stained feature was graded on a scale from 0 to 4: 0, absent and appears normal; 1, less than 25%; 2, 25 to less than 50%; 3, 50 to less than 75%; 4, 75% or higher. The four scores were added together and the total score used as the lung injury score for the sections. An investigator blinded to group assignment conducted all assessments.

### 2.5 Immunohistochemistry (IHC)

For IHC, the left lung sections (5-µm thickness) were deparaffinized with xylene, rehydrated in a series of graded concentrations of ethanol, and then underwent antigen repair using citrate buffer (pH 6.0). All lung tissue sections were treated with 3% H202 for 25 min to remove endogenous peroxidase, followed by blocking with 3% BSA for 30 min at room temperature. Subsequently, the sections were incubated overnight at 4°C with Myeloperoxidase Rabbit pAb (MPO, 1:500, GB11224, Servicebio) or Anti-CD68 Rabbit pAb (CD68, 1:200, GB113109, Servicebio). After washing, the sections were incubated with HRP-conjugated Goat Anti-Rabbit secondary antibody (1:500; GB23303, Servicebio) for 50 min at room temperature. DAB (G1212, Servicebio) was used for chromogenic staining, with the staining time controlled under a microscope until positive staining appeared as brown-yellow, followed by washing the sections with running water to stop the staining. Subsequent steps included counterstaining with hematoxylin for 3 min, differentiation in HCl-alcohol for 20 s, incubation with 1% ammonia for 30 s to regain the blue color, rinsing with running water, dehydrating in alcohol, clearing in xylene, and sealing with neutral gum.

### 2.6 8-hydroxyguanosine (8-OHdG) immunofluorescence

Immunofluorescence staining for 8-hydroxyguanosine (8-OHdG) was performed. Deparaffinized and rehydrated lung sections were incubated with FITC anti-DNA/RNA damage antibody (Ab183393, Abcam) at a 1:200 dilution overnight at 4°C, followed by FITC goat anti-Mouse (green) at a 1:50 dilution. DAPI (G1012, Servicebio) was used for nuclear counterstaining. The number of total and 8-OHdG-positive cells per high power field was counted in 10 different random fields for each coded slide using the ImageJ program (NIH, Bethesda, MD).

### 2.7 Real-time quantitative polymerase chain reaction (qPCR)

Lung tissue total RNA was extracted in NucleoZOL (740404.200; Macherey-Nagel, Germany). The quality and integrity of extracted RNA were evaluated using Nanodrop Specthophotometer. The cDNA was synthesized using HiScript III All-in-one RT SuperMix (R333-01, Vazyme, China). The housekeeping gene actin beta (Actb) was used as a reference. All primers used in this study were mRNA specific and designed for qPCR (Bio-Rad CFX Manager, United States) using ChamQ Universal SYBR qPCR Master Mix (Q711-02, Vazyme, China). Primers details are presented in Table 1. Melting-curve analysis was conducted for all PCR runs. Gene expression before and after the HBO treatment was compared using the 2^−ΔΔCT^ (fold change) relative quantification method. The control group value and associated variability were expected to be close to 1.

**TABLE 1:** Nomenclature, gene information, and mRNA primer characteristics.

Gene symbol	GENE full name	NCBI GENE id	Sequence (5′→3′)
Actb	actin beta	11461	GGCTGTATTCCCCTCCATCG
			CCAGTTGGTAACAATGCCATGT
Il6	interleukin 6	16193	TAGTCCTTCCTACCCCAATTTCC
			TTGGTCCTTAGCCACTCCTTC
Ccl2	chemokine (C-C motif) ligand 2	20296	TTAAAAACCTGGATCGGAACCAA
			GCATTAGCTTCAGATTTACGGGT
Ccl3	chemokine (C-C motif) ligand 3	20302	TTCTCTGTACCATGACACTCTGC
			CGTGGAATCTTCCGGCTGTAG
Cxcl5	chemokine (C-X-C motif) ligand 5	20311	TCCAGCTCGCCATTCATGC
			TTGCGGCTATGACTGAGGAAG
Cxcl10	chemokine (C-X-C motif) ligand 10	15945	CCAAGTGCTGCCGTCATTTTC
			GGCTCGCAGGGATGATTTCAA

Top sequence reflects the forward primer, and bottom sequence reflects the reverse primer.

### 2.8 Western blot assay (WB)

Lung tissues were lysed using RIPA Lysis buffer (P0013B; Beyotime, China) containing protease and phosphatase inhibitor cocktail (dilution 1:100, 78442; Thermo Fisher Scientific, United States) for protein samples. The protein concentration was measured using the BCA method (23227; Thermo Fisher Scientific, United States). The protein samples were mixed with 5× sample buffer (MB01015; GenScript, United States) and subjected to sodium dodecyl sulfate-polyacrylamide gel electrophoresis (SDS-PAGE). The electrophoresed protein samples were then transferred onto PVDF membranes. Following blocking with 5% fat-free milk for 1 h at room temperature, the membranes were incubated overnight at 4°C with the following antibodies: cadherin 5 (VE Cadherin, VE-Cad, dilution 1:1,000; ab33168; Abcam), tight junction protein 1 (Zona occludens 1, ZO-1, dilution 1:5,000, 21773-1-AP; Proteintech), intercellular adhesion molecule 1 (ICAM1) (dilution 1:1,000, ab222736; Abcam), vascular cell adhesion molecule 1 (VCAM1) (dilution 1:1,000, ab134047; Abcam), and beta actin (β-actin) (dilution 1:5,000, 20536-1-AP; Proteintech). Subsequently, the membranes were probed using Image Studio (Ver 5.2, LI-COR) after incubation with corresponding secondary antibodies (dilution 1:5,000, SA00001-1, SA00001-2, Proteintech) for 1 h at room temperature.

### 2.9 Enzyme-linked immunosorbent assay (ELISA)

Lung samples were snap-frozen in liquid nitrogen and stored at −80°C until use. For cytokine levels, lung tissues were measured using the commercially available ELISA kits (BioTNT, China) for mouse Tnf, Il1b, Il6, Cxcl1, and Ccl2, according to the manufacturer’s protocol. The total protein of lung tissue homogenates was determined using the BCA method. The levels of cytokines in lung tissue were expressed as pg per mg of total protein in the lung tissue homogenate (pg/mg prot).

### 2.10 Statistical analyses

Statistical analyses were performed with Graphpad Prism 8.0.1 (Graphpad Software, La Jolla, CA). Results are checked the distribution normality by the Shapiro-Wilk test, for normally distributed data are presented as mean ± standard deviation (SD), and non-normally distributed data are presented as median and interquartile range (IQR, p25-p75). Ordinary one-way ANOVA were used to compare the measurement data among groups. A *p*-value less than 0.05 was considered statistically significant.

## 3 Results

### 3.1 Lung histopathology in response to different durations of HBO exposure

As shown in Figure 1A, five groups of mice were exposed to room air or HBO for 2, 4, 6, or 8 h, respectively, all mice survived without obvious abnormal symptoms. As shown in Figure 1C, H&E staining revealed localized bronchial and alveolar dilation in lung tissue of the 4-h HBO exposure group. In the 6-h HBO exposure group, there was atrophy of the bronchi and alveoli surrounding the dilated bronchi, along with a significant increase in alveolar hemorrhage and inflammatory cell infiltration. To further verify the increased infiltration of inflammatory cells, neutrophils and macrophages were labeled with MPO and CD68 respectively (Figures 1D, E). Pathological scores of lung tissue showed that, compared to the room air control group, the injury scores of the 6-h and 8-h HBO exposure groups were significantly higher (4.350 ± 1.464, 5.667 ± 1.586 vs. 0.933 ± 0.859; *p* = 0.0461, *p* = 0.0031, respectively) (Figure 1B).

### 3.2 Oxidative DNA damage in lung tissue in response to different durations of HBO exposure

**FIGURE 2:**
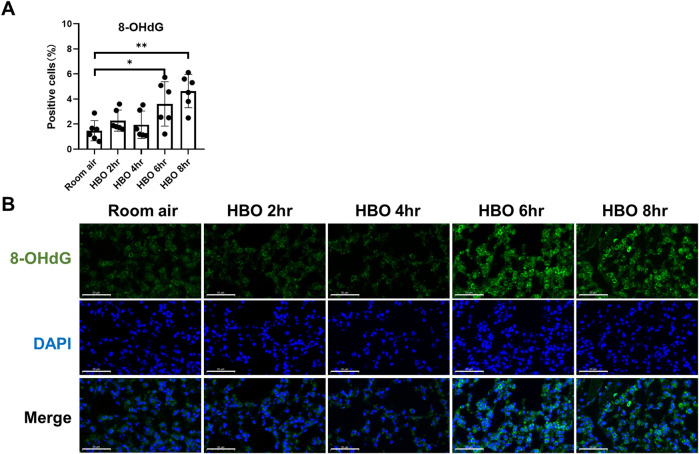
Oxidative DNA damage in lung tissue in response to different durations of HBO exposure. **(A)** Quantitative analysis of 8-OHdG in lung tissue sections as described in the Methods section. n = 6, ∗*p* ≤ 0.05, ∗∗*p* ≤ 0.01. **(B)** Representative images of 8-OHdG staining to assess DNA oxidative damage in lung tissues. Scale bars: 50 µm.

Continuous *in vivo* HBO exposure leads to the accumulation of reactive oxygen species (ROS), resulting in the formation of 8-hydroxy-2′-deoxyguanosine (8-OHdG), a biomarker for secondary metabolites of oxidative DNA damage. Immunofluorescence analysis revealed a significant increase in the number of 8-OHdG-positive cells in the lung tissues of the 6- and 8-h HBO groups compared to the air control group (3.606 ± 1.772, 4.640 ± 1.332 vs. 1.468 ± 0.802; *p* = 0.0406, *p* = 0.0012) (Figures 2A, B). Apoptosis in lung tissue, another important manifestation of HBO-induced ALI, was determined using the TdT-mediated dUTP Nick-End Labeling (TUNEL). The number of positive cells in the HBO-exposed group was minimal and did not significantly differ from the control group (Supplementary Figure S2).

### 3.3 Alveolar-capillary barrier in response to different durations of HBO exposure

**FIGURE 3:**
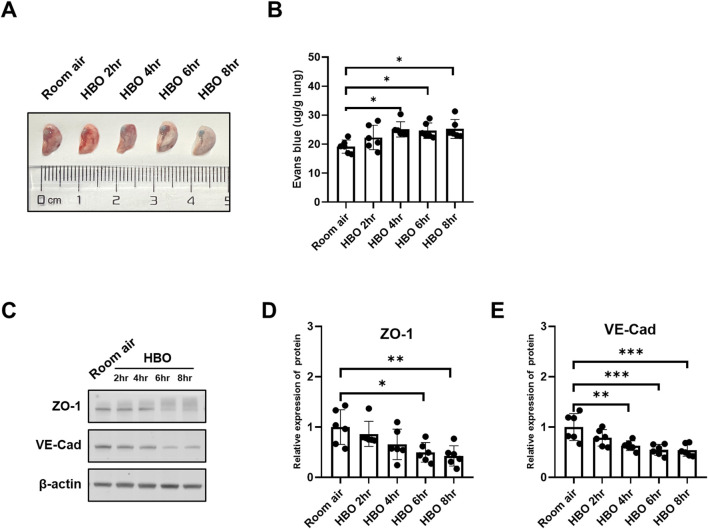
Alveolar-capillary barrier in response to different durations of HBO exposure. **(A)** Representative image depicting extravasation of Evans-Blue dye in the lung. **(B)** Quantitative results showing the concentration of Evans-Blue dye in lung tissue. **(C)** Representative WB images of ZO-1 and VE-Cad in mouse lung samples; β-actin used as the loading control. **(D, E)** Quantitative graph of WB bands of ZO-1 and VE-Cad. n = 6, ∗*p* ≤ 0.05, ∗∗*p* ≤ 0.01, ∗∗∗*p* ≤ 0.001.

The results indicated a tendency of increased pulmonary vascular permeability with longer durations of HBO exposure, as depicted in Figures 3A, B. The 4-, 6-, and 8-h HBO-exposed groups exhibited significantly increased pulmonary vascular permeability compared to the air control group [24.21 (23.58, 26.24), 24.66 ± 2.661, 23.83 (23.02, 27.85) vs. 19.17 ± 2.265 μg/g lungs; *p* = 0.0200, *p* = 0.0361, and *p* = 0.0166, respectively].

To further investigate the extent of alveolar-capillary barrier damage induced by HBO, bronchoalveolar lavage fluid (BALF) was collected to measure total protein content, and WB was conducted to assess the expression levels VE-Cad and ZO-1. Compared to the Room air control group, The BALF total protein was significantly higher in the HBO 6 h group (Supplementary Figure S1). As depicted in Figures 3C–E, the expression of VE-Cad and ZO-1 decreased as the duration of HBO exposure increased. In the 4-h, 6-h, and 8-h HBO exposed groups, the expression levels of VE-Cad were significantly lower compared to the Room air group (0.6308 ± 0.09636, 0.5470 ± 0.1081, 0.5432 ± 0.1208 vs. 1.0000 ± 0.2670; *p* = 0.0052, *p* = 0.0006, and *p* = 0.0005, respectively) (Figures 3F, H). ZO-1 expression was significantly reduced in the 6-h and 8-h HBO groups (0.4983 ± 0.1944, 0.4279 ± 0.2017 vs. 1.000 ± 0.3443; *p* = 0.0230, *p* = 0.0077, respectively).

### 3.4 Inflammatory factors and chemokines in lung tissues in response to different durations of HBO exposure

ALI caused by HBO exposure can lead to increased expression of inflammatory factors and adhesion molecules. We used WB to detect the expression changes of ICAM1 and VCAM1 in lung tissue, and qPCR to detect the expression changes of genes related to inflammatory cytokines and chemokines in lung tissues.

**FIGURE 4:**
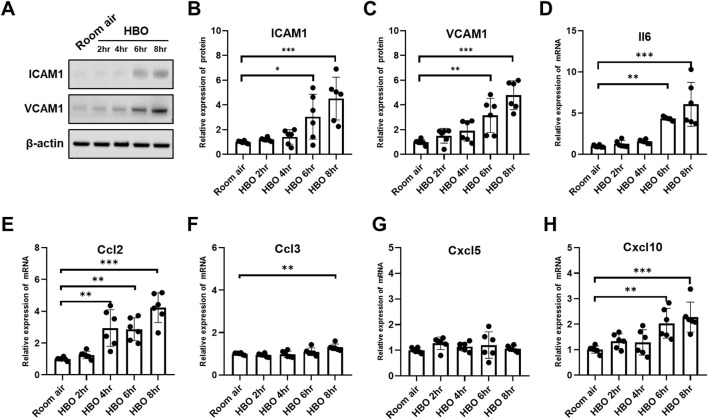
Inflammatory factors and chemokines in lung tissues in response to different durations of HBO exposure. **(A)** Western blot analysis of ICAM1 and VCAM1 in mouse lung samples; β-actin used as the loading control. **(B, C)** Quantitative graph of Western blot analysis. **(D–H)** Expression of inflammatory cytokines and chemokines in lung tissues detected by qPCR as fold change. n = 6, ∗*p* ≤ 0.05, ∗∗*p* ≤ 0.01, ∗∗∗*p* ≤ 0.001.

The expression of both the ICAM1 and VCAM1 proteins increased with the extension of HBO exposure duration (Figure 4A). In the 6- and 8-h HBO groups, the expression of ICAM1 and VCAM1 were significantly higher than the air control group (ICAM1: 3.049 ± 1.815, 4.517 ± 1.729 vs. 1.000 ± 0.099; *p* < 0.01; VCAM1: 3.152 ± 1.379, 4.790 ± 1.167 vs. 1.000 ± 0.217; *p* < 0.01) (Figures 4B, C). The lung tissues of the 6-h and 8-h HBO groups exhibited significantly higher expression of Il6 and Cxcl10 compared to the air control group (Il6: 4.366 ± 0.2261, 6.081 ± 2.661 vs. 1.000 ± 0.1393; *p* < 0.01; Cxcl10: 2.032 ± 0.5861, 2.278 ± 0.5889 vs. 1.000 ± 0.1407, *p* < 0.01) (Figures 4D, H). Ccl2 expression was significantly increased after 4 h of HBO exposure (2.947 ± 1.152, 2.868 ± 0.7163, 4.229 ± 0.9360 vs. 1.000 ± 0.1019; *p* < 0.01) (Figure 4E). Ccl3 expression was significantly elevated in the 8-h HBO group (1.302 ± 0.1547 vs. 1.000 ± 0.05024, *p* = 0.0043) (Figure 4F). However, HBO had no significant effect on the mRNA expression of Cxcl5 (Figure 4G). These data suggest that alveolar-capillary barrier dysfunction and inflammatory response increased when exposed to HBO for more than 6 h.

### 3.5 Changes in histopathology during the recovery period after exposure to HBO

**FIGURE 5:**
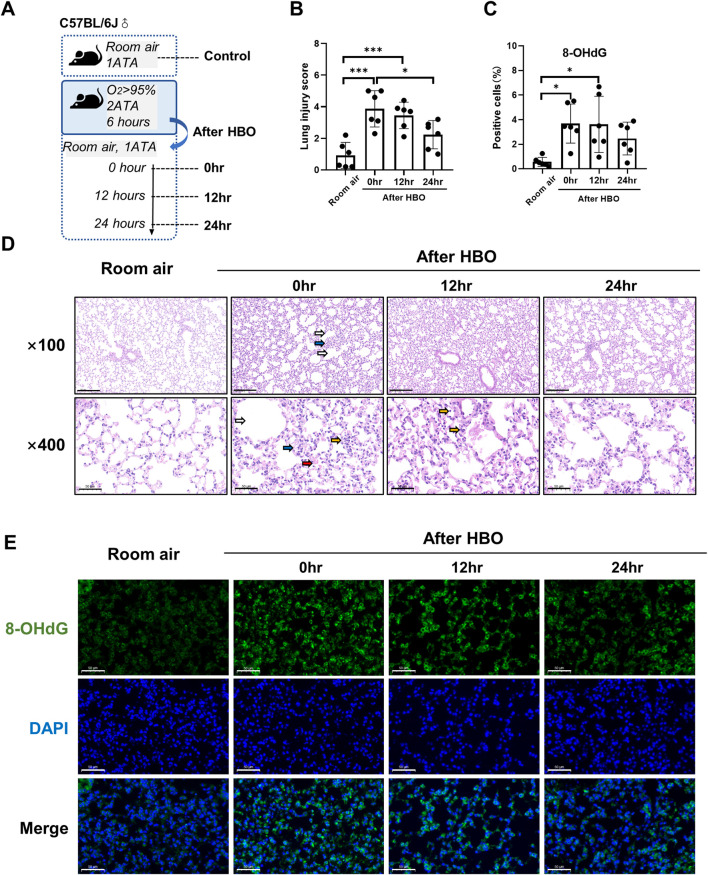
Histopathology and oxidative DNA damage in lung tissue during the recovery period after exposure to HBO. **(A)** Experimental scheme. In the control group (Room air), mice were placed in the hyperbaric chamber at atmospheric pressure with air, while the experimental groups were exposed to 2 ATA and ≥95% oxygen for 6 h. Lung tissue samples were collected from experimental groups at 0 h (0 h), 12 h (12 h), and 24 h (24 h) after the exposure. **(B)** Assessment of lung injury scores for H&E-stained images. **(C)** Quantitative analysis of 8-OHdG in lung tissue sections as described in the Methods section. **(D)** Representative H&E-stained images of mouse lung sections. Yellow arrows: infiltration of alveolar and interstitial inflammatory cells; red arrows: alveolar and interstitial hemorrhage; blue arrows: atelectasis; white arrows: alveolar dilation. Scale bars: 200 µm (100×) and 50 µm (400×). **(E)** Representative images of 8-OHdG staining to assess DNA oxidative damage in lung tissues. Scale bars: 50 μm. n = 6, ∗*p* ≤ 0.05, ∗∗*p* ≤ 0.01, ∗∗∗*p* ≤ 0.001.

To observe the recovery patterns of lung tissue in animals after high-pressure oxygen exposure. We exposed three groups of mice to 2 ATA hyperoxia for 6 h, and then collected lung tissue at 0, 12, and 24 h post-exposure for analysis (Figure 5A). The control group was exposed to air. After 12 and 24 h of 6-h-HBO exposure, all mice still survived without obvious abnormal symptoms. Through H&E staining of lung tissues, the dilatation and atrophy of bronchial and alveolar, alveolar hemorrhage, and inflammatory cell infiltration were still evident after 12 h of 6-h-HBO exposure, after 24 h, these injuries improved. The lung injury score of the 0 h and 12 h after HBO groups were significantly higher than the Room air group (3.867 ± 1.148, 3.450 ± 0.841 vs. 0.917 ± 0.828; *p* = 0.0001, *p* = 0.0008, respectively), and the lung injury score of the 24 h after HBO group was significantly lower than the 0h after HBO group (2.233 ± 0.894 vs. 3.867 ± 1.148; *p* = 0.0316) (Figure5B, D).

### 3.6 Changes in oxidative DNA damage in lung tissue during the recovery period after exposure to HBO

The expression levels of 8-OHdG and the number of apoptotic cells in lung tissue were measured during the recovery period following exposure to HBO. The results showed that the number of 8-OHdG-positive cells in lung tissue was significantly higher at 0 h and 12 h after removal from the high oxygen environment compared to the room air control group (3.679 ± 1.593, 3.613 ± 2.291 vs 0.567 ± 0.371, *p* = 0.0122, *p* = 0.0144), but there was no significant difference between 0 h, 12 h, and 24 h (Figures 5C, E). TUNEL staining showed that there were still very few positive cells in lung tissue at each time point during the recovery period, and there was no significant difference compared to the air control group (Supplementary Figure S2).

### 3.7 Changes in alveolar-capillary barrier during the recovery period after exposure to HBO

**FIGURE 6:**
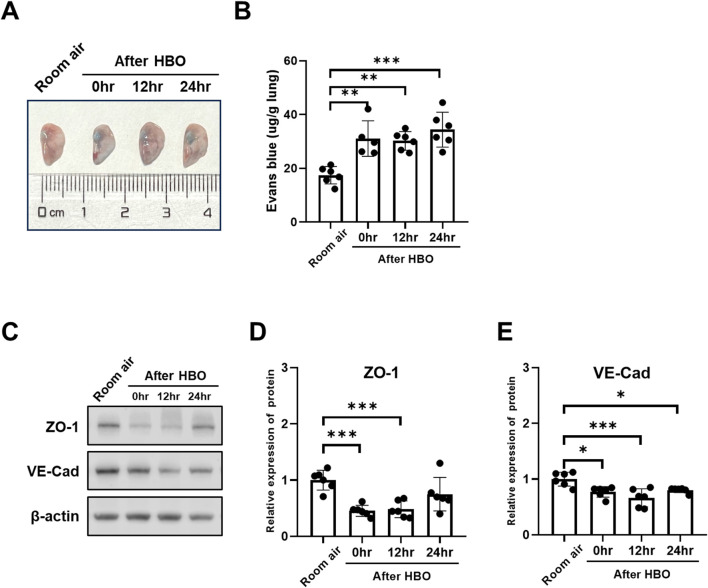
Alveolar-capillary barrier during the recovery period after exposure to HBO. **(A)** Representative image depicting extravasation of Evans-Blue dye in the lung. **(B)** Quantitative results showing the concentration of Evans-Blue dye in lung tissue. **(C)** Representative WB images of ZO-1 and VE-Cad in mouse lung samples; β-actin used as the loading control. **(D, E)** Quantitative graph of WB bands of ZO-1 and VE-Cad. n = 6, ∗*p* ≤ 0.05, ∗∗*p* ≤ 0.01, ∗∗∗*p* ≤ 0.001.

EB in lung levels showed less pronounced trend after HBO, as the Figures 6A, B showed, compared with the Room air group, the EB in lungs of the 0 h, 12 h, and 24 h after HBO groups were significantly higher (31.06 ± 6.604, 30.21 ± 3.461, 34.41 ± 6.473 vs. 17.44 ± 3.220 μg/g lung; *p* = 0.0016, *p* = 0.0019, *p* < 0.001, respectively), but no significant difference between 0 h, 12 h, and 24 h.

The expression of VE-Cad and ZO-1 also showed less pronounced trend after HBO, as the Figures 6C–E showed, compared with the Room air group, the expression levels of VE-Cad were significantly lower after HBO 0 h, 12 h, and 24 h (0.7698 ± 0.0942, 0.6659 ± 0.1611, 0.8030 ± 0.0452 vs. 1.000 ± 0.1248; *p* = 0.0166, *p* = 0.0003, *p* = 0.0343, respectively) (Figures 4F, H). After 0 h and 12 h, the expression levels of ZO-1 were significantly lower (0.4561 ± 0.0987, 0.4818 ± 0.1484 vs. 1.000 ± 0.1774; *p* = 0.0005, *p* = 0.0009, respectively), after 24 h of HBO, there seemed to be a partial recovery in the expression levels of ZO-1, because there was no significant difference compared with Room air group (0.7507 ± 0.2975 vs. 1.000 ± 0.1774; *p* = 0.1528).

### 3.8 Changes in inflammatory response in lung tissue during the recovery period after exposure to HBO

**FIGURE 7:**
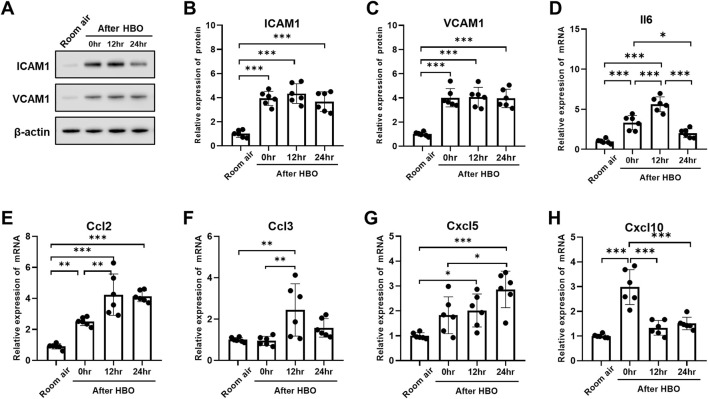
Inflammatory response in lung tissue during the recovery period after exposure to HBO. **(A)** Western blot analysis of ICAM1 and VCAM1 in mouse lung samples; β-actin used as the loading control. **(B, C)** Quantitative graph of Western blot analysis. **(D–H)** Expression of inflammatory cytokines and chemokines in lung tissues detected by qPCR as fold change. n = 6, ∗*p* ≤ 0.05, ∗∗*p* ≤ 0.01, ∗∗∗*p* ≤ 0.001.

The expression of the ICAM1 and VCAM1 after HBO groups were significantly higher than the air control group (ICAM1: 3.938 ± 0.587, 4.337 ± 0.818, 3.663 ± 0.814 vs. 1.000 ± 0.289; *p* < 0.0001; VCAM1: 4.007 ± 0.758, 4.052 ± 0.819, 3.953 ± 0.764 vs. 1.000 ± 0.179; *p* < 0.0001). However, during the recovery period at 0, 12, and 24 h after exposure to HBO, there was no significant change in the expression of ICAM1 and VCAM1 proteins in lung tissue (Figures 7A–C).

Compared to 0 h, the mRNA expression of Il6, Ccl2, and Ccl3 significantly increased at 12 h (Il6: 5.624 ± 0.9272 vs. 3.328 ± 0.8934, *p* < 0.0001; Ccl2: 4.240 ± 1.332 vs. 2.511 ± 0.2626, *p* = 0.0021; Ccl3: 2.442 ± 1.267 vs. 0.9443 ± 0.2209, *p* = 0.0057) (Figures 7D–F). Cxcl5 showed no significant change at 0 h, compared with air control (1.824 ± 0.7388 vs. 1.000 ± 0.1221, *p* = 0.1318), but significantly increased after 12 h (2.107 ± 0.6638 vs. 1.000 ± 0.1221, *p* = 0.0462) (Figure 7G). Compared to 0 h and 12 h, Il6 significantly decreased after 24 h (2.017 ± 0.5523 vs. 3.328 ± 0.8934, 2.017 ± 0.5523 vs. 5.624 ± 0.9272, *p* < 0.01) (Figure 7D). Compared to 0 h, the expression of Cxcl10 also significantly decreased after 12 and 24 h (1.331 ± 0.2962, 1.507 ± 0.2529 vs. 2.981 ± 0.7037, *p* < 0.0001) (Figure 7H). However, the expression level of Ccl3 at 24 h showed no difference compared to the air control group (1.573 ± 0.4477 vs. 1.000 ± 0.1069, *p* = 0.4823) (Figure 7F).

**FIGURE 8:**
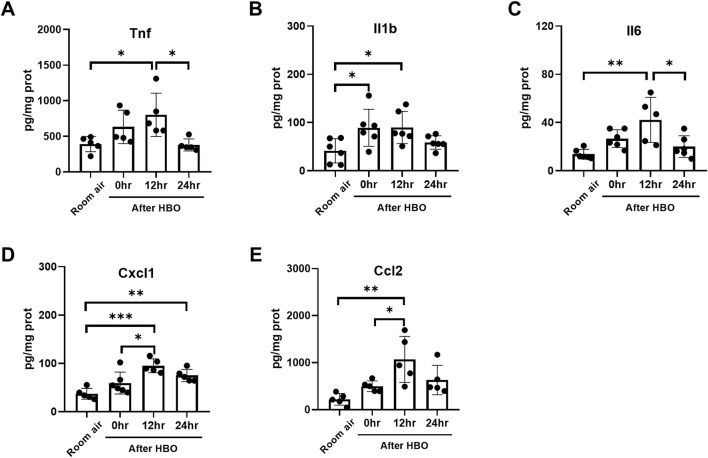
Inflammatory response in lung tissue during the recovery period after exposure to HBO. **(A–E)** Expression of inflammatory cytokines and chemokines in lung tissues detected by ELISA. n = 5–6, ∗*p* ≤ 0.05, ∗∗*p* ≤ 0.01, ∗∗∗*p* ≤ 0.001.

Furthermore, the changes in the protein levels of inflammatory and chemotactic factors in lung tissue were further examined using ELISA. The results showed that the protein levels of inflammatory and chemotactic factors reached a peak at 12 h after HBO exposure. Compared to the Room air group, there were significant increases in the protein levels of Tnf, Il1b, Il6, Cxcl1, and Ccl2 at 12 h after HBO exposure (Tnf: 801.4 ± 304.7 vs. 390.6 ± 107.2 pg/mg prot, *p* = 0.0265; Il1b: 89.68 ± 33.35 vs. 41.28 ± 25.09 pg/mg prot, *p* = 0.0437; Il6: 42.18 ± 18.73 vs. 13.78 ± 4.012 pg/mg prot, *p* = 0.0016; Cxcl1: 95.02 ± 14.31 vs. 37.06 ± 11.31 pg/mg prot, *p* = 0.0002; Ccl2: 1,068 ± 490.4 vs. 220.2 ± 124.5 pg/mg prot, *p* = 0.0022). At 24 h after the HBO exposure, these levels began to decrease. Compared to 12 h after HBO exposure, there were significant reductions in the protein levels of TNF and IL-6 at 24 h (Tnf: 378.9 ± 84.84 vs. 801.4 ± 304.7 pg/mg prot, *p* = 0.0221; Il6: 20.03 ± 8.998 vs. 42.18 ± 18.73 pg/mg prot, *p* = 0.0136). Compared to the room air group, the protein levels of Tnf, Il1b, Il6, and CCL2 at 24 h after HBO exposure had recovered and showed no significant statistical difference (Tnf: 378.9 ± 84.84 vs. 390.6 ± 107.2 pg/mg prot, *p* = 0.9997; Il1b: 58.63 ± 14.08 vs. 41.28 ± 25.09 pg/mg prot, *p* = 0.7360; Il6: 20.03 ± 8.998 vs. 13.78 ± 4.012 pg/mg prot, *p* = 0.7430; Ccl2: 630.5 ± 314.2 vs. 220.2 ± 124.5 pg/mg prot, *p* = 0.1828) (Figures 8A–E). These results indicate that the inflammatory response continues to develop after the end of high-pressure oxygen exposure, reaching its peak at 12 h and recovering after 24 h.

## 4 Discussion

The study explored the immediate effects of 2ATA hyperbaric oxygen (HBO) exposure on lung tissue at different times, and the recovery pattern of lung tissue after HBO exposure. The main findings are as follows: 2ATA hyperbaric oxygen exposure can cause significant lung tissue damage after 6 h, mainly manifested as alveolar expansion, atelectasis, inflammatory cell infiltration, and hemorrhage. Compared with lung damage caused by normobaric oxygen, the time of damage onset is significantly shortened. Lung damage continues to progress after leaving the high-oxygen environment. The study found that in mice exposed to 2ATA HBO for 6 h, the inflammatory response and alveolar-capillary barrier damage in lung tissue continued to progress after leaving the high-oxygen environment, peaking at 12 h and beginning to recover after 24 h. This is inconsistent with the commonly held belief that lung damage can be alleviated immediately upon leaving the high-oxygen environment. The alveolar-capillary barrier is the most susceptible to damage and recovers the slowest. The results showed that after 4 h of 2ATA oxygen exposure, there was a significant increase in pulmonary vascular endothelial permeability and changes in endothelial markers. These damages persisted 24 h after leaving the high-oxygen environment, but the pathological changes in lung tissue showed significant improvement at this time, with oxidative DNA damage and the expression of inflammatory and chemotactic factors significantly reduced. These results can provide a reference for understanding the pathogenesis and progression of lung injury caused by high-pressure oxygen and for finding corresponding intervention targets.

In this study, we suggest that HBO exposure of 6 h or more causes ALI in mice. In an official American Thoracic Society (ATS) workshop report (Kulkarni et al., 2022), it is proposed that ALI is characterized by the following four key pathophysiologic features: 1) histological evidence of tissue injury, 2) alteration of the alveolar-capillary barrier, 3) presence of an inflammatory response, and 4) physiologic dysfunction. Animal models must document alterations of at least three of the four domains to qualify as “experimental ALI.” Based on ATS recommendations for ALI animal models, although evidence of partial alveolar-capillary barrier damage (increased Evans blue leakage and decreased VE-cad) was observed in the HBO 4-h group, no significant changes were observed in histological scores and inflammatory responses; therefore, we did not define the HBO 4-h group as HBO-ALI. Additionally, we calculated the value of K for based on the pulmonary oxygen toxicity index (POT index) formula K = t^2^ × PO_2_^4.57^. The calculated K value for 6 h of 2ATA HBO was approximately 894, which is higher than the threshold of 799 associated with causing POT (Arieli, 2023). Therefore, we have chosen a duration of 6-h HBO in the second part to evaluate the recovery of HBO-ALI.

Our research findings further confirm that the alveolar-capillary barrier is most sensitive to hyperbaric oxygen (HBO) exposure. Significant increase in EB leakage was observed as early as 4 h after HBO exposure, suggesting damage to endothelial gap junctions or alveolar epithelium. VE-Cad significantly decreased after 4 h of HBO exposure, and ZO-1 also showed a significant decrease after 6 h of HBO. ICAM1 and VCAM1 began to rise 4 h after HBO exposure, with significant increases at 6–8 h, preceding changes in the expression of inflammatory factors. As pulmonary adhesion molecules, ICAM1 and VCAM1, as lung adhesion molecules, bind to the endothelial surface and are involved in leukocyte infiltration through the ECs and into the lungs; their elevation is considered an early event in endothelial dysfunction. These results further suggest that alveolar-capillary barrier is the earliest to respond to oxygen toxicity. The damaged epithelium and endothelium can increase the retention of platelets and neutrophils in the pulmonary microvasculature, promoting the subsequent occurrence and development of inflammatory responses (Meng et al., 2015; Amarelle et al., 2021).

What is interesting is that we also observed that alveolar-capillary barrier was the last to recover after detachment from a high-oxygen environment. Our results indicate that 24 h after the cessation of HBO, lung tissue damage scores and the expression of inflammatory factors had essentially returned to baseline levels. However, there was still a significant increase in EB leakage, and the expression level of VE-Cad remained decreased. These findings suggest that the intercellular junctions of the pulmonary vascular endothelium were still in a damaged state. After damage to the alveolar microvascular barrier function, the restoration of the gas exchange barrier requires multiple steps, including the proliferation of quiescent endothelial cells to replace damaged and apoptotic cells, as well as the re-establishment of adherens junctions (AJ) between microvascular endothelial cells to form a new barrier (Yuan et al., 2013). Therefore, this may be the reason why alveolar-capillary barrier remains impaired after detachment from a high-oxygen environment. This research result is also consistent with clinical changes. Currently, DL_CO_ (lung diffusion capacity for carbon monoxide) is considered a sensitive index of complete recovery from pulmonary oxygen toxicity (van Ooij et al., 2011). DL_CO_ is an indicator of gas diffusion function and is a sensitive indicator of damage to the blood-gas barrier. Our study suggests that the delayed recovery of alveolar-capillary barrier damage might be one of the reasons for the slow recovery of DL_CO_ following exposure to high oxygen.

Excessive ROS from high oxygen levels directly damage cells, considering the transient nature of ROS that makes direct detection challenging, we inferred the presence of ROS by assessing DNA oxidative damage. Oxidative damage to DNA usually focuses on oxidation of guanine to 8-oxo-7,8-dihydro-2′-deoxyguanosine (8OHdG, or 8-oxodG). The amount of 8OHdG measured in DNA is the balance between the rate of oxidation and that of repair (Murphy et al., 2022). We observed significant DNA oxidative damage in lung tissue exposed to HBO. Results indicate that DNA damage in lung tissue significantly increases after 6 h of HBO exposure, which is markedly faster than lung damage caused by normobaric high oxygen. Nagato et al. (2012) detected oxidative stress in lung tissue after exposing mice to normobaric high oxygen (100% oxygen) for 12 h. These findings suggest again that lung damage induced by HBO progresses quicker than normobaric oxygen, consistent with our previous research (Xiaochen et al., 2014).

The study not only examined the onset of ALI damage due to 2ATA HBO exposure but also observed the outcome of pulmonary injury after leaving the high oxygen environment. ALI induced by hyperoxia may be due to direct damage from excess ROS production, or by exacerbating inflammation (Min et al., 2012). Our findings indicate that HBO exposure significantly increases the expression levels of inflammatory and chemotactic cytokines, including Il6, Ccl2, Ccl3, and Cxcl10, consistent with previous research on normobaric hyperoxia-induced ALI (Li et al., 2011; Entezari et al., 2014; Dias-Freitas et al., 2016; Ning et al., 2020). However, under HBO, significant increases in these factors are seen at 6 h, whereas it takes at least 24 h under normobaric conditions, with histological damage appearing at 48 h (Nagato et al., 2012). These results confirm that HBO-induced ALI requires a shorter exposure time and develops faster than normobaric oxygen. Furthermore, after leaving the HBO environment, lung tissue damage continues to progress, with inflammation persisting for at least 12 h and beginning to recover after 24 h. Inflammatory cytokines remained elevated at 12 h after leaving the HBO environment and began to recover at 24 h, including Il6, Ccl2, Ccl3, Cxcl5, and Cxcl10.

Inflammation is critical to the development of ALI, we speculate that excessive inflammation plays a critical role in determining the outcome of HBO-ALI. Il6 is one of the master regulators of inflammatory processes, facilitating neutrophil recruitment to the lungs (Florentin et al., 2021). A decrease in its level (Chen et al., 2021) or gene expression (Chernikov et al., 2024) is associated with a weakening of ALI. Our results revealed the same trend between the expression of Il6 and the severity of lung injury. Based on this, we speculate that Il6 could be a potential therapeutic target for the management of HBO-ALI. In addition, we observed increased expression of pro-inflammatory cytokines Ccl2, Ccl3, and Cxcl10 in HBO-ALI lung tissue, and excessive cytokines may exacerbate the progression of lung injury (Major et al., 2020). Interestingly, Ccl3 did not show a significant increase immediately after 6 h of HBO exposure, but it significantly elevated 12 h post-exposure. CCL3, a ligand produced by immune cells such as neutrophils, DCs, and monocytes, facilitates the migration of CCR1^+^ neutrophil subpopulations (Charmoy et al., 2010; Repeke et al., 2010; Sokol and Luster, 2015; Liu et al., 2017). *In vitro* data suggest that Il6 promotes the expression of Ccl3 (Holloman et al., 2023). In our study, the elevation of Ccl3 occurred later than the infiltration of inflammatory cells and the increase of Il6 in lung tissue, which may be due to Ccl3 being primarily secreted by neutrophils and monocytes/macrophages. Its elevation could be a marker of heightened hyperimmune responses in lung tissue (Maus et al., 2002; Hegde et al., 2015; Rajarathnam et al., 2019; Johansson and Kirsebom, 2021). Cxcl5 may relate to delayed damage or repair in HBO-ALI. We observed that Cxcl5 did not change significantly immediately after 6 h of HBO exposure but increased markedly at 12 and 24 h post-exposure. Produced by resident lung cells (such as pulmonary epithelial cells and platelets during lung inflammation) (Jeyaseelan et al., 2005), Cxcl5 plays a key role in controlling chemokine clearance (Mei et al., 2010) and can negatively regulate neutrophil influx into the lungs by affecting the concentration of other chemokines in the bloodstream (Koltsova and Ley, 2010; Mei et al., 2010; Guo et al., 2021). We hypothesize that Cxcl5 may suppress further inflammation in the HBO-ALI inflammatory response. Further work is needed to confirm this.

In this study, there were no significant changes in lung tissue TUNEL staining. The apoptosis shown by TUNEL was a common method for detecting DNA fragmentation that results from apoptosis signaling cascades (Yao X. et al., 2019). In lung injury caused by normobaric hyperoxia, the apoptosis of pulmonary epithelial cells is considered to be an important characteristic of hyperoxia-induced lung injury (Cao et al., 2016; Yao H. et al., 2019), but there is still controversy over cell damage caused by HBO. According to current research, whether apoptosis is induced by HBO is related to the extent of DNA damage. Schönrock et al. found that HBO exposure significantly induced oxidative stress and DNA damage in osteoblast lineage cells (primary human osteoblasts, HOBs, and the osteogenic tumor cell line SAOS-2), without a significant increase in apoptosis (Schönrock et al., 2023). Stefan (Weber et al., 2009) and others exposed Jurkat-T-cells to environmental air or oxygen at 1-3ATA and found that HBO induced apoptosis in the cells through the mitochondrial pathway. The human promyelocytic cell line HL-60 (Ganguly et al., 2002), the T-lymphoblastic cell line JURKAT, and the B-lymphoblastic cell line CCRF-SB (De Bels et al., 2020) were also induced to undergo apoptosis by HBO. It is currently believed that the mode of high-level oxidative stress caused by HBO exposure affects the induction of apoptosis in specific cells. HBO-induced DNA damage is mainly related to the formation of single-strand breaks (single-strand break/single-stranded DNA Damage, SSB) rather than double-strand breaks (double-strand break/double-stranded DNA damage, DSB), and such damage can be effectively repaired. The triggered repair and adaptation patterns can prevent long-term DNA damage (Schönrock et al., 2023), thereby avoiding the occurrence of cellular apoptosis. The exposure time for hyperoxia-induced apoptosis in lung tissue cells is often as long as 24 h (Cao et al., 2016) or even longer (Yao H. et al., 2019), while our HBO intervention lasted no more than 8 h. Therefore, we speculate that short-term HBO exposure, although it quickly results in DNA damage, is the easily repairable SSB. This damage can be effectively repaired and triggers adaptive mechanisms such as the increased expression of Hemeoxygenase-1 (HO-1) (known to increase in oxidative stress and have an anti-apoptotic effect (Loboda et al., 2008)), thus preventing more severe cell damage, such as apoptosis. Of course, making inferences based solely on current research is not rigorous enough, and further research is needed to verify it.

In summary, our study systematically observed the progression and outcomes of ALI induced by HBO. The degree of lung injury caused by HBO increased in a time-dependent manner, with pulmonary tissue damage occurring after 4 h of exposure to 2ATA HBO. Pulmonary alveolar-capillary barrier is the most sensitive to high oxygen levels, sustaining the earliest damage and also recovering the slowest. The lung injury caused by 6 h of exposure to 2ATA HBO was mainly characterized by alveolar-capillary barrier and inflammatory damage. Inflammatory responses in lung tissue peaked 12 h after leaving the high-oxygen environment and began to recover after 24 h, with no significant cellular apoptosis observed. This study is focused solely on the phenomenon of lung injury induced by hyperoxia and does not delve into the mechanisms of pathogenesis, which is a limitation of this research. However, this study systematically examines the disease process and recovery stages of hyperoxia-induced lung injury, which can aid in understanding the pathological process. Additionally, it provides a reference for the selection of appropriate interventions and timing in the future, which is the focus of our ongoing work.

## Data Availability

The raw data supporting the conclusions of this article will be made available by the authors, without undue reservation.

## References

[B1] Alva R. Mirza M. Baiton A. Lazuran L. Samokysh L. Bobinski A. (2023). Oxygen toxicity: cellular mechanisms in normobaric hyperoxia. Cell Biol. Toxicol. 39 (1), 111–143. 10.1007/s10565-022-09773-7 36112262 PMC9483325

[B40] Amarelle L. Quintela L. Hurtado J. Malacrida L. (2021). Hyperoxia and Lungs: What We Have Learned From Animal Models. Front Med (Lausanne) 8, 606678. 10.3389/fmed.2021.606678 33768102 PMC7985075

[B2] Arieli R. (2023). The pulmonary oxygen toxicity index. Respir. Physiol. Neurobiol. 315, 104114. 10.1016/j.resp.2023.104114 37460079

[B3] Bao X. C. Fang Y. Q. You P. Zhang S. Ma J. (2014). Protective role of peroxisome proliferator-activated receptor β/δ in acute lung injury induced by prolonged hyperbaric hyperoxia in rats. Respir. Physiol. Neurobiol. 199, 9–18. 10.1016/j.resp.2014.04.004 24780550

[B4] Bao X. C. Mao A. R. Fang Y. Q. Fan Y. H. Wang F. F. Ma J. (2018). Simvastatin decreases hyperbaric oxygen-induced acute lung injury by upregulating eNOS. Am. J. Physiol. Lung Cell Mol. Physiol. 314 (2), L287–L297. 10.1152/ajplung.00520.2016 29074491

[B5] Bennett M. H. Lehm J. P. Mitchell S. J. Wasiak J. (2010). Recompression and adjunctive therapy for decompression illness: a systematic review of randomized controlled trials. Anesth. Analg. 111 (3), 757–762. 10.1213/ANE.0b013e3181cdb081 20332190

[B6] Cao Y. Zhang D. Moon H. G. Lee H. Haspel J. A. Hu K. (2016). MiR-15a/16 regulates apoptosis of lung epithelial cells after oxidative stress. Mol. Med. 22, 233–243. 10.2119/molmed.2015.00136 27257854 PMC5023515

[B7] Capellier G. Maupoil V. Boussat S. Laurent E. Neidhardt A. (1999). Oxygen toxicity and tolerance. Minerva Anestesiol. 65 (6), 388–392. 10394807

[B41] Charmoy M. Brunner-Agten S. Aebischer D. Auderset F. Launois P. Milon G. (2010). Neutrophil-derived CCL3 is essential for the rapid recruitment of dendritic cells to the site of Leishmania major inoculation in resistant mice. PLoS Pathog 6 (2), e1000755. 10.1371/journal.ppat.1000755 20140197 PMC2816696

[B8] Chen I. C. Wang S. C. Chen Y. T. Tseng H. H. Liu P. L. Lin T. C. (2021). Corylin ameliorates LPS-induced acute lung injury via suppressing the MAPKs and IL-6/STAT3 signaling pathways. Pharm. (Basel) 14 (10), 1046. 10.3390/ph14101046 PMC8537250 34681270

[B9] Chernikov I. V. Bachkova I. K. Sen'kova A. V. Meschaninova M. I. Savin I. A. Vlassov V. V. (2024). Cholesterol-modified anti-il6 siRNA reduces the severity of acute lung injury in mice. Cells 13 (9), 767. 10.3390/cells13090767 38727303 PMC11083178

[B10] De Bels D. Tillmans F. Corazza F. Bizzari M. Germonpre P. Radermacher P. (2020). Hyperoxia alters ultrastructure and induces apoptosis in leukemia cell lines. Biomolecules 10 (2), 282. 10.3390/biom10020282 32059539 PMC7072400

[B11] Demchenko I. T. Welty-Wolf K. E. Allen B. W. Piantadosi C. A. (2007). Similar but not the same: normobaric and hyperbaric pulmonary oxygen toxicity, the role of nitric oxide. Am. J. Physiol. Lung Cell Mol. Physiol. 293 (1), L229–L238. 10.1152/ajplung.00450.2006 17416738

[B12] Dias-Freitas F. Metelo-Coimbra C. Roncon-Albuquerque R. Jr. (2016). Molecular mechanisms underlying hyperoxia acute lung injury. Respir. Med. 119, 23–28. 10.1016/j.rmed.2016.08.010 27692143

[B13] Entezari M. Javdan M. Antoine D. J. Morrow D. M. Sitapara R. A. Patel V. (2014). Inhibition of extracellular HMGB1 attenuates hyperoxia-induced inflammatory acute lung injury. Redox Biol. 2, 314–322. 10.1016/j.redox.2014.01.013 24563849 PMC3926109

[B14] Florentin J. Zhao J. Tai Y. Y. Vasamsetti S. B. O'Neil S. P. Kumar R. (2021). Interleukin-6 mediates neutrophil mobilization from bone marrow in pulmonary hypertension. Cell Mol. Immunol. 18 (2), 374–384. 10.1038/s41423-020-00608-1 33420357 PMC8027442

[B15] Ganguly B. J. Tonomura N. Benson R. M. Osborne B. A. Granowitz E. V. (2002). Hyperbaric oxygen enhances apoptosis in hematopoietic cells. Apoptosis 7 (6), 499–510. 10.1023/a:1020686908831 12370492

[B16] Guo L. Li N. Yang Z. Li H. Zheng H. Yang J. (2021). Role of CXCL5 in regulating chemotaxis of innate and adaptive leukocytes in infected lungs upon pulmonary influenza infection. Front. Immunol. 12, 785457. 10.3389/fimmu.2021.785457 34868067 PMC8637413

[B17] Han C. H. Guan Z. B. Zhang P. X. Fang H. L. Li L. Zhang H. M. (2018). Oxidative stress induced necroptosis activation is involved in the pathogenesis of hyperoxic acute lung injury. Biochem. Biophys. Res. Commun. 495 (3), 2178–2183. 10.1016/j.bbrc.2017.12.100 29269294

[B47] Hegde V. L. Singh U. P. Nagarkatti P. S. Nagarkatti M. (2015). Critical Role of Mast Cells and Peroxisome Proliferator-Activated Receptor γ in the Induction of Myeloid-Derived Suppressor Cells by Marijuana Cannabidiol *In Vivo* . J Immunol 194 (11), 5211–5222. 10.4049/jimmunol.1401844 25917103 PMC4433789

[B45] Holloman B. L. Cannon A. Wilson K. Singh N. Nagarkatti M. Nagarkatti P. (2023). Characterization of Chemotaxis-Associated Gene Dysregulation in Myeloid Cell Populations in the Lungs during Lipopolysaccharide-Mediated Acute Lung Injury. J Immunol 210 (12), 2016–2028. 10.4049/jimmunol.2200822 37163318 PMC10615667

[B18] Jeyaseelan S. Manzer R. Young S. K. Yamamoto M. Akira S. Mason R. J. (2005). Induction of CXCL5 during inflammation in the rodent lung involves activation of alveolar epithelium. Am. J. Respir. Cell Mol. Biol. 32 (6), 531–539. 10.1165/rcmb.2005-0063OC 15778492 PMC2715322

[B49] Johansson C. Kirsebom F. C. M. (2021). Neutrophils in respiratory viral infections. Mucosal Immunol 14 (4), 815–827. 10.1038/s41385-021-00397-4 33758367 PMC7985581

[B19] Koltsova E. K. Ley K. (2010). The mysterious ways of the chemokine CXCL5. Immunity 33 (1), 7–9. 10.1016/j.immuni.2010.07.012 20643334

[B20] Kulkarni H. S. Lee J. S. Bastarache J. A. Kuebler W. M. Downey G. P. Albaiceta G. M. (2022). Update on the features and measurements of experimental acute lung injury in animals: an official American thoracic society workshop report. Am. J. Respir. Cell Mol. Biol. 66 (2), e1–e14. 10.1165/rcmb.2021-0531ST 35103557 PMC8845128

[B21] Li L. F. Yang C. T. Huang C. C. Liu Y. Y. Kao K. C. Lin H. C. (2011). Low-molecular-weight heparin reduces hyperoxia-augmented ventilator-induced lung injury via serine/threonine kinase-protein kinase B. Respir. Res. 12 (1), 90. 10.1186/1465-9921-12-90 21726460 PMC3136419

[B44] Liu J. Pang Z. Wang G. Guan X. Fang K. Wang Z. (2017). Advanced Role of Neutrophils in Common Respiratory Diseases. J Immunol Res, 6710278. 10.1155/2017/6710278 28589151 PMC5447318

[B22] Loboda A. Jazwa A. Grochot-Przeczek A. Rutkowski A. J. Cisowski J. Agarwal A. (2008). Heme oxygenase-1 and the vascular bed: from molecular mechanisms to therapeutic opportunities. Antioxid. Redox Signal 10 (10), 1767–1812. 10.1089/ars.2008.2043 18576916

[B23] Major J. Crotta S. Llorian M. McCabe T. M. Gad H. H. Priestnall S. L. (2020). Type I and III interferons disrupt lung epithelial repair during recovery from viral infection. Science 369 (6504), 712–717. 10.1126/science.abc2061 32527928 PMC7292500

[B46] Maus U. von Grote K. Kuziel W. A. Mack M. Miller E. J. Cihak J. (2002). The role of CC chemokine receptor 2 in alveolar monocyte and neutrophil immigration in intact mice. Am J Respir Crit Care Med 166 (3), 268–273. 10.1164/rccm.2112012 12153956

[B24] Mei J. Liu Y. Dai N. Favara M. Greene T. Jeyaseelan S. (2010). CXCL5 regulates chemokine scavenging and pulmonary host defense to bacterial infection. Immunity 33 (1), 106–117. 10.1016/j.immuni.2010.07.009 20643340 PMC3748840

[B39] Meng F. Mambetsariev I. Tian Y. Beckham Y. Meliton A. Leff A. (2015). Attenuation of lipopolysaccharide-induced lung vascular stiffening by lipoxin reduces lung inflammation. Am J Respir Cell Mol Biol 52 (2), 152–161. 10.1165/rcmb.2013-0468OC 24992633 PMC4370244

[B25] Min J. H. Codipilly C. N. Nasim S. Miller E. J. Ahmed M. N. (2012). Synergistic protection against hyperoxia-induced lung injury by neutrophils blockade and EC-SOD overexpression. Respir. Res. 13 (1), 58. 10.1186/1465-9921-13-58 22816678 PMC3441354

[B26] Murphy M. P. Bayir H. Belousov V. Chang C. J. Davies K. J. A. Davies M. J. (2022). Guidelines for measuring reactive oxygen species and oxidative damage in cells and *in vivo* . Nat. Metab. 4 (6), 651–662. 10.1038/s42255-022-00591-z 35760871 PMC9711940

[B27] Nagato A. C. Bezerra F. S. Lanzetti M. Lopes A. A. Silva M. A. Porto L. C. (2012). Time course of inflammation, oxidative stress and tissue damage induced by hyperoxia in mouse lungs. Int. J. Exp. Pathol. 93 (4), 269–278. 10.1111/j.1365-2613.2012.00823.x 22804763 PMC3444983

[B28] Ning K. Guan Z. B. Lu H. T. Zhang N. Sun X. J. Liu W. W. (2020). Lung macrophages are involved in lung injury secondary to repetitive diving. J. Zhejiang Univ. Sci. B 21 (8), 646–656. 10.1631/jzus.B1900687 32748580 PMC7445088

[B48] Rajarathnam K. Schnoor M. Richardson R. M. Rajagopal S. (2019). How do chemokines navigate neutrophils to the target site: Dissecting the structural mechanisms and signaling pathways. Cell Signal 54), 69–80. 10.1016/j.cellsig.2018.11.004 30465827 PMC6664297

[B42] Repeke C. E. Ferreira S. B. Jr Claudino M. Silveira E. M. de Assis G. F. Avila-Campos M. J. (2010). Evidences of the cooperative role of the chemokines CCL3, CCL4 and CCL5 and its receptors CCR1+ and CCR5+ in RANKL+ cell migration throughout experimental periodontitis in mice. Bone 46 (4), 1122–1130. 10.1016/j.bone.2009.12.030 20053385

[B29] Schönrock N. Tillmans F. Sebens S. Kähler W. Klapa S. Rieger B. (2023). Analysis of single- and double-stranded DNA damage in osteoblastic cells after hyperbaric oxygen exposure. Antioxidants (Basel) 12 (4), 851. 10.3390/antiox12040851 37107226 PMC10135236

[B43] Sokol C. L. Luster A. D. (2015). The chemokine system in innate immunity. Cold Spring Harb Perspect Biol 7 (5). 10.1101/cshperspect.a016303 PMC4448619 25635046

[B30] Taabazuing C. Y. Hangasky J. A. Knapp M. J. (2014). Oxygen sensing strategies in mammals and bacteria. J. Inorg. Biochem. 133, 63–72. 10.1016/j.jinorgbio.2013.12.010 24468676 PMC4097052

[B31] van Ooij P. J. van Hulst R. A. Houtkooper A. Sterk P. J. (2011). Differences in spirometry and diffusing capacity after a 3-h wet or dry oxygen dive with a PO(2) of 150 kPa. Clin. Physiol. Funct. Imaging 31 (5), 405–410. 10.1111/j.1475-097X.2011.01034.x 21771262

[B32] Wang J. Li R. Peng Z. Hu B. Rao X. Li J. (2020). HMGB1 participates in LPS-induced acute lung injury by activating the AIM2 inflammasome in macrophages and inducing polarization of M1 macrophages via TLR2, TLR4, and RAGE/NF-κB signaling pathways. Int. J. Mol. Med. 45 (1), 61–80. 10.3892/ijmm.2019.4402 31746367 PMC6889921

[B33] Wang R. Li Q. Wu P. Ren K. Li Y. Wang Y. (2024). Fe-capsaicin nanozymes attenuate sepsis-induced acute lung injury via NF-κB signaling. Int. J. Nanomedicine 19, 73–90. 10.2147/ijn.S436271 38187907 PMC10771734

[B34] Weber S. U. Koch A. Kankeleit J. Schewe J.-C. Siekmann U. Stüber F. (2009). Hyperbaric oxygen induces apoptosis via a mitochondrial mechanism. Apoptosis 14 (1), 97–107. 10.1007/s10495-008-0280-z 19052874

[B35] Xiaochen B. Yiqun F. Dan L. Fangfang W. Jun M. Pu Y. (2014). Observation on the lung injury induced by normobaric and hyperbaric oxygen exposures in experimental rats. Chin J Naut Med and Hyperbar Med (01), 43–46. 10.3760/cma.j.issn.1009-6906.2014.01.011

[B36] Yao H. Gong J. Peterson A. L. Lu X. Zhang P. Dennery P. A. (2019a). Fatty acid oxidation protects against hyperoxia-induced endothelial cell apoptosis and lung injury in neonatal mice. Am. J. Respir. Cell Mol. Biol. 60 (6), 667–677. 10.1165/rcmb.2018-0335OC 30571144 PMC6543740

[B37] Yao X. Dong G. Zhu Y. Yan F. Zhang H. Ma Q. (2019b). Leukadherin-1-Mediated activation of CD11b inhibits LPS-induced pro-inflammatory response in macrophages and protects mice against endotoxic shock by blocking LPS-TLR4 interaction. Front. Immunol. 10, 215. 10.3389/fimmu.2019.00215 30809230 PMC6379471

[B38] Yuan J. Guo Q. Qureshi A. R. Anderstam B. Eriksson M. Heimbürger O. (2013). Circulating vascular endothelial growth factor (VEGF) and its soluble receptor 1 (sVEGFR-1) are associated with inflammation and mortality in incident dialysis patients. Nephrol. Dial. Transpl. 28 (9), 2356–2363. 10.1093/ndt/gft256 23828162

