# Supplementary material for: Hyperbaric oxygen-induced acute lung injury: A mouse model study on pathogenic characteristics and recovery dynamics
Source: Front Physiol. 2024 Oct 18;15:1474933. doi: 10.3389/fphys.2024.1474933 (PMC11527661; doi:10.3389/fphys.2024.1474933)
Supplement: Supplementary file 1 [file Table1.DOCX]

Supplementary Material

# Supplementary materials and methods

## Bronchoalveolar lavage fluid (BALF)

After the trachea was exposed, a 20-G catheter was inserted into the trachea for lavage. Cold PBS (1 ml) was instilled into the mouse lungs followed by gentle aspiration repeated three times. All of the BALF was centrifuged and the supernatant was collected to measure total protein content using BCA assay.

## TUNEL

TUNEL assay was performed on deparaffinized and rehydrated sections. The sections were stained with TMR (red) TUNEL apoptosis detection kit (G1502, Servicebio) following the manufacturer's instructions. The nuclear counterstain was done with DAPI (G1012, Servicebio) in the dark. The number of total and TUNEL-positive cells per high power field was counted in 10 different random fields for each coded slide using the Image J program. (NIH, Bethesda, MD).

# Supplementary figures

## Supplementary figure 1

**Supplementary Figure 1.** Quantification of total protein in bronchoalveolar lavage fluid. Compared to the Room air control group, The BALF total protein was significantly higher in the HBO 6hr group (733.5 ± 112.4 vs. 384.6 ± 73.24 µg/mL, p < 0.001). n=6, * *p* ≤ 0.05, ** *p* ≤ 0.01, *** *p* ≤ 0.001.

##
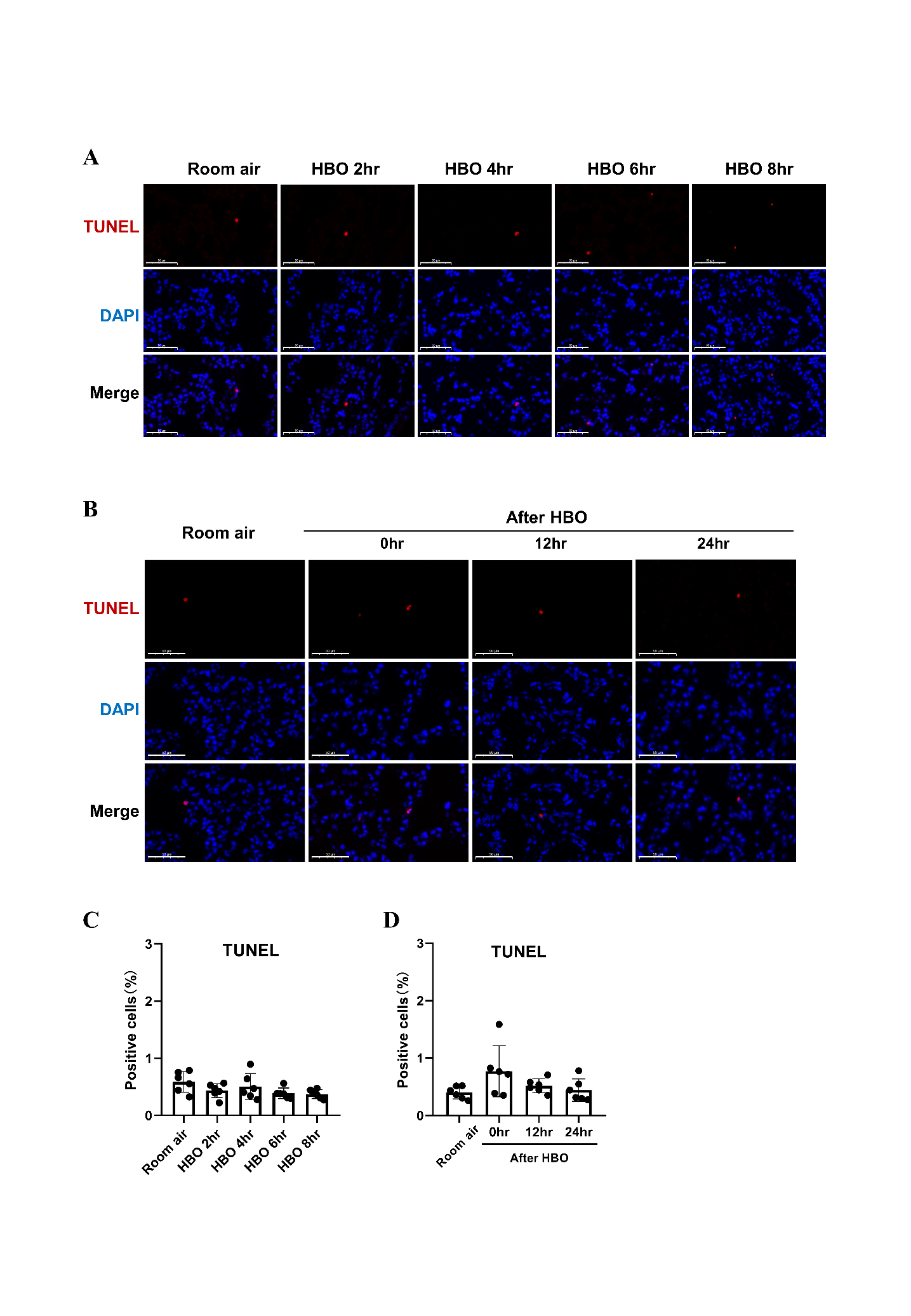
Supplementary figure 2

**Supplementary figure 2.** TdT-mediated dUTP Nick-End Labeling (TUNEL) staining of lung tissues. (A) Representative images in response to different durations of HBO exposure. Scale bars: 50 µm. (B) Representative images during the recovery period after exposure to HBO. Scale bars: 50 µm. (C-D) Quantitative analysis of TUNEL in lung tissue sections as described in the Methods section. (n = 6, *p* ＞ 0.05).

##
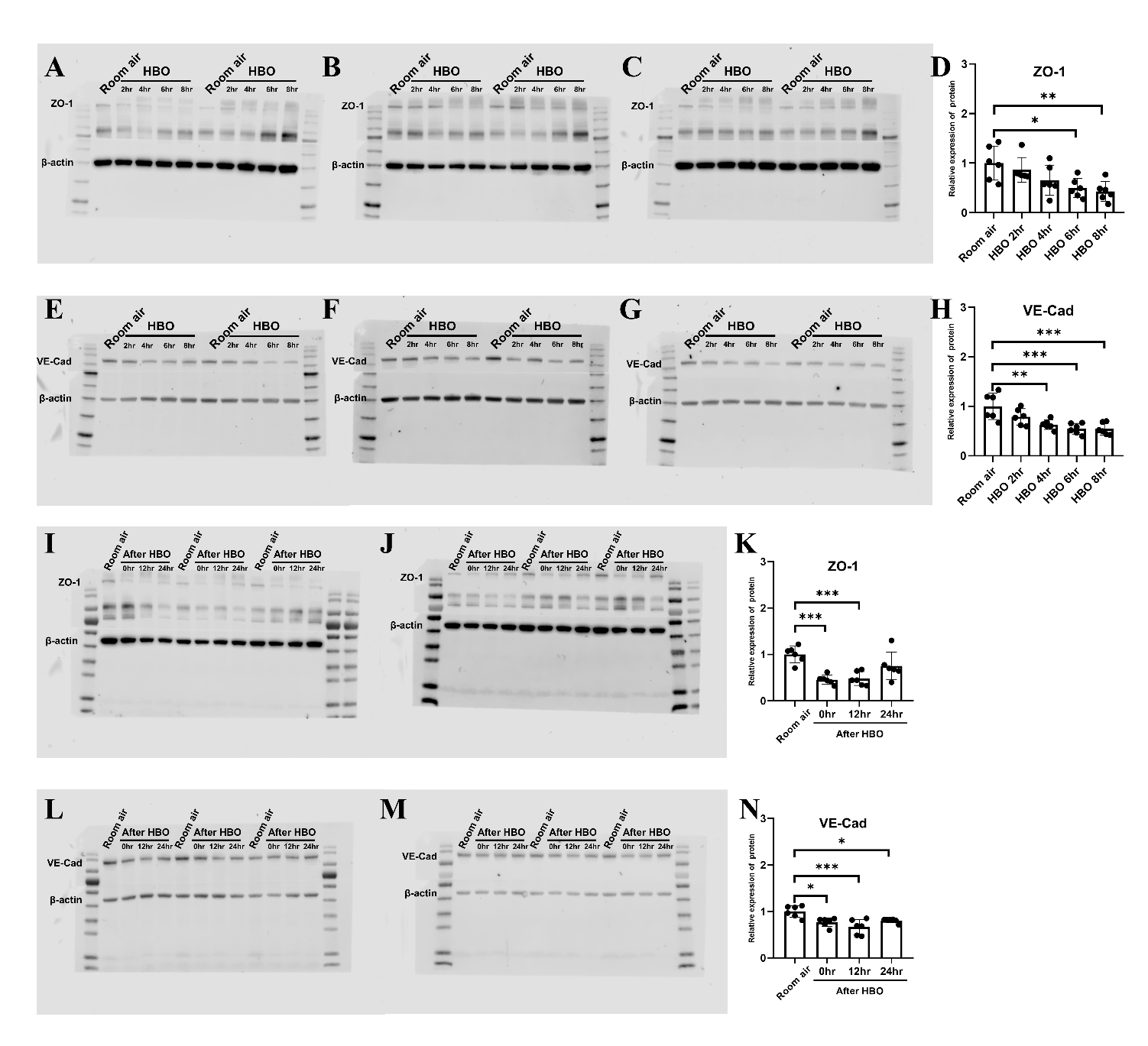
Supplementary Figure 3

**Supplementary Figure 3.** The original WB images of ZO-1 and VE-Cad.

##
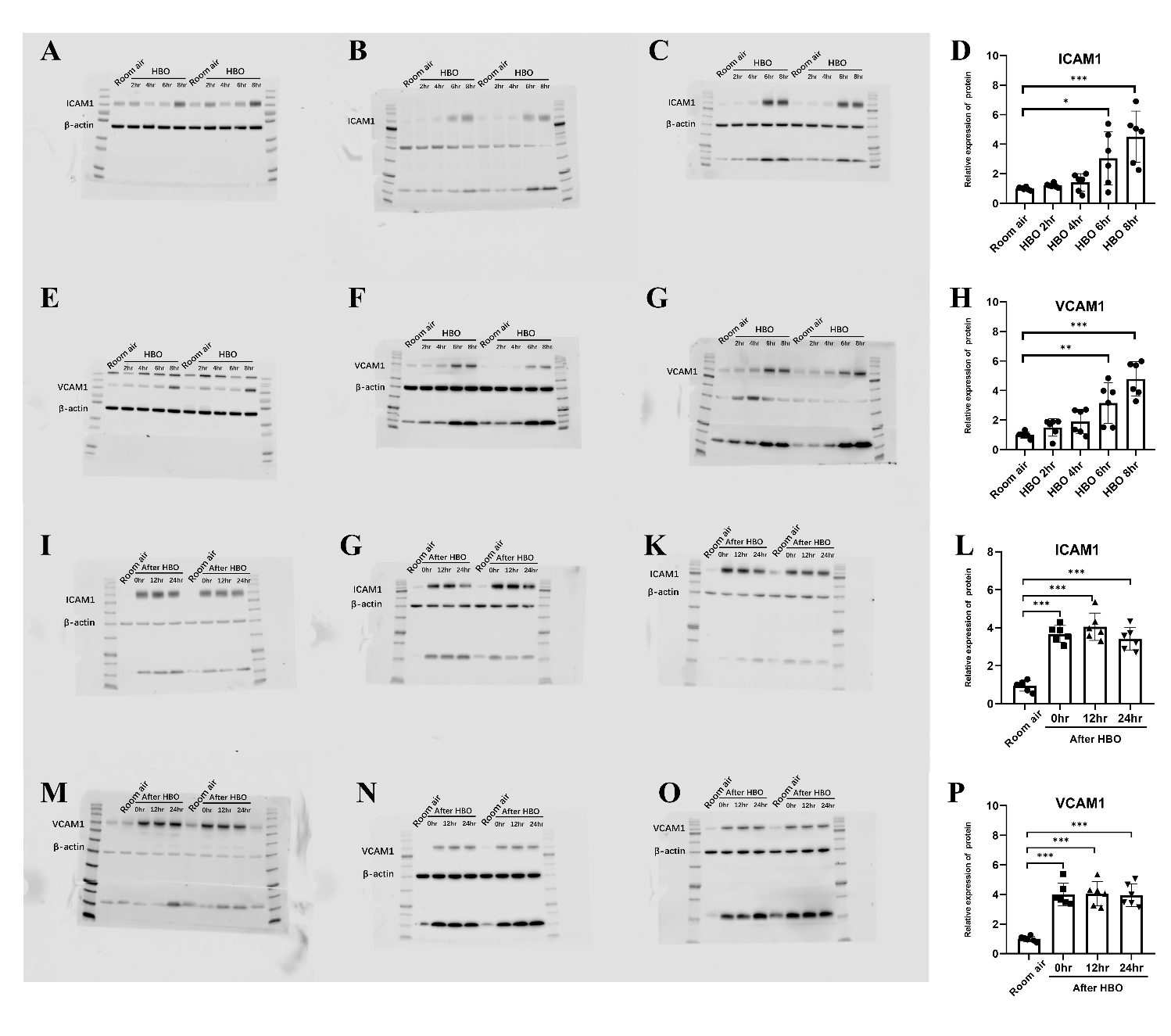
Supplementary Figure 4

**Supplementary Figure 4.** The original WB images of ICAM1 and VCAM1. The loading order of samples for B and F is the same, as well as the loading order of samples for C and G.
